# Anxiety Status and Influencing Factors of Rural Residents in Hunan During the Coronavirus Disease 2019 Epidemic: A Web-Based Cross-Sectional Survey

**DOI:** 10.3389/fpsyt.2020.564745

**Published:** 2020-11-24

**Authors:** Yi Zhang, Yi-ping Chen, Jianjian Wang, Yanhong Deng, Dezhen Peng, Liping Zhao

**Affiliations:** ^1^Xiangya Nursing School, Central South University, Changsha, China; ^2^The Second Xiangya Hospital, Central South University, Changsha, China

**Keywords:** corona virus disease 2019, rural residents, anxiety, Hunan (South China), COVID-19

## Abstract

**Objective:** To explore the status quo of anxiety and its influencing factors among rural residents in Hunan Province during the coronavirus disease 2019 epidemic, and to provide an effective basis for prevention of and intervention for anxiety symptoms among rural residents.

**Methods:** Convenience sampling was used. An online questionnaire was distributed to Hunan rural residents through the questionnaire star platform from February 26–29, 2020. The general data and anxiety of Hunan rural residents were investigated, and the data were analyzed using SPSS 18.0.

**Results:** The mean Self-Rating Anxiety Scale score of 179 rural residents in Hunan was 40.93 ± 9.36. Based on the cutoff criteria, 32 residents had anxiety, including 26 with mild anxiety, five with moderate anxiety, and one with severe anxiety. The detection rate of anxiety was 17.88%. Self-rated health status, level of concern about the epidemic, and self-rated impact of the epidemic on one's life were the factors influencing the anxiety score of rural residents in Hunan (*P* < 0.05).

**Conclusion:** During the coronavirus disease 2019 epidemic, the detection rate of anxiety in rural residents in Hunan was higher than that of the general population in China. The relevant departments should pay attention to the mental health of rural residents and implement targeted mental health prevention and intervention measures during the epidemic situation.

## Introduction

Since December 2019, coronavirus disease 2019 (COVID-19) epidemics have appeared around the world, starting in Wuhan, Hubei Province, China (1). As of October 4, 2020, the total number of confirmed cases worldwide was 34,804,348, with 1,030,738 cumulative associated deaths (2). The total number of confirmed cases in China was 85,470, with 4,634 deaths (3). It has had a considerable impact and caused psychological strain among Chinese people. Without doubt, the COVID-19 epidemic is a global public health problem that poses a serious threat to the global society, economy, and human health. The cognitive—phenomenological—transactional model proposes that stress is generated through the specific relationship between the individual and the environment. The individual continually recognizes and evaluates stimuli in a stressful environment, and undergoes physical and psychological changes to adapt to the needs of the environment (4). Therefore, in the face of an epidemic, different groups of people will have different levels of anxiety, fear, helplessness, and even impulsive and irritating behaviors (5). Many studies have shown that mental health problems could occur in both medical staff and severe acute respiratory syndrome (SARS) survivors during the SARS epidemic (6–9). Previous studies of Middle East respiratory syndrome also reported similar results (10, 11). Researchers have conducted many surveys on the psychological status of people during the COVID-19 epidemic, but most of them have concentrated on special groups such as patients with COVID-19 (12, 13), medical staff (14–16), or susceptible groups such as the elderly (17), children (18), and students (19). There are few studies on the psychological status of residents, especially rural residents. Rural areas have become extremely challenging for epidemic prevention and control because of various factors such as shortages of medical resources, insufficient public protection capabilities, and population migration caused by the return of migrant workers to their hometown (20). Previous research found that rural residents have poorer mental health compared to urban residents (21), and anxiety is one of the main problems affecting the mental health of rural residents (22). Hubei was the first and most severely affected province in the epidemic. As its neighboring province, Hunan Province is more likely to be exposed to suspected or infected cases than other regions, which increases residents' psychological pressure and anxiety. This study investigated the anxiety status of rural residents in Hunan during the COVID-19 epidemic and analyzed its influencing factors, intending to provide a scientific basis for effective psychological intervention for rural residents in Hunan.

## Methods

### Study Design and Sample

A cross-sectional study was conducted among rural residents in rural areas of Hunan Province, China, from February 26–29, 2020. The inclusion criteria were as follows: (a) living in the rural areas of Hunan Province during the COVID-19 epidemic; (b) aged 18 years or older; (c) proficient in WeChat and online questionnaires; and (d) were conscious and volunteered for the study. The exclusion criteria were as follows: participants with psychosis or severe mental disorders and inadequate communication ability.

Ethical approval was obtained from the ethics committee of Central South University (No: E202020) before data collection.

### Measures

*Socio-demographic characteristics* were measured using a self-designed questionnaire, including gender, age, occupation, residence, marital status, relevant knowledge of COVID-19 (understanding relevant knowledge or not, channels of knowledge acquisition), health status (temperature, self-rated health status, history of contact with Wuhan within the previous half month, and history of exposure to potentially infected people), level of concern about the epidemic, and self-rated impact of the epidemic on one's life.

*Anxiety status* was determined using the self-rating anxiety scale (SAS) (23). The scale consists of 20 items and aims to assess respondents' subjective symptoms. Each item was evaluated on a four-point Likert scale ranging from 1 (“little or none of the time”) to 4 (“most of the time”), with higher scores indicating higher levels of anxiety. The Cronbach's alpha for the total scale was 0.864, and it has good reliability and validity, and has been widely used in clinical research (24). According to the Chinese norm (25), the standard score has a cutoff value of 50, 50 to 59 represents mild anxiety, 60 to 68 represents moderate anxiety, and 69 points and above represents severe anxiety.

### Statistical Analysis

#### Sample Size

We selected 18 possible influencing factors through a literature review. According to the principle that the sample size should be 5 to 10 times the number of independent variables, the estimated minimum sample size was 180; considering a likely attrition rate of 10% and sampling error, the final required sample size was 200.

#### Quality Control

This survey used an online questionnaire. The questionnaire could only be submitted after it had been completed and each IP address could only answer once to avoid repeated answers. In addition, real-time background monitoring was performed to ensure data reliability. To prevent possible bias, a uniform guideline was used on the front page of the questionnaire to explain the completion requirements.

#### Data Analysis

Data analysis was performed using SPSS 18.0. Results are expressed as mean ± standard deviation (X ± S) or number (%). First, descriptive analyses were conducted to describe the demographic characteristics, relevant knowledge of COVID-19, and health status in rural residents in Hunan during the epidemic. Second, *t*-tests and one-way analysis of variance were used to analyze the anxiety levels of residents with different characteristics. Third, a *t*-test was used to compare the anxiety scores between rural residents in Hunan during the epidemic and different populations in different periods. Fourth, hierarchical regression analysis was performed to explore potential factors influencing residents' anxiety in Hunan during the COVID-19 epidemic. *P*-values < 0.05 were considered statistically significant (2-sided tests).

## Results

We collected 200 questionnaires in the rural areas of 45 townships in 15 regions in Hunan Province. After checking each item one by one, a total of 21 questionnaires with high consistency of answers or answering time of <150 s were excluded, and 179 valid questionnaires were obtained. The response rate of the questionnaire was 89.5%.

As shown in Table 1, most participants were living with their spouse or family and only a few lived alone (5.6%). Most participants had relevant knowledge of COVID-19 (93.9%), and official platforms were their main channels for acquiring knowledge (76.5%). Most participants expressed concern about this epidemic (93.9%), and only 3.9% of them self-rated that the epidemic did not affect their lives. Among them, 32 of the 179 participants (17.88%) had anxiety; specifically, 26 participants had mild anxiety, five participants had moderate anxiety, and one participant had severe anxiety.

**Table 1:** Analysis of anxiety levels of people with different characteristics in Hunan rural residents during the epidemic of COVID-19.

**Variable**		***N***	**%**	**Mean (SD)**		***F*/*t***	***P***
Sex	①Male ②Female	55 124	30.7 69.3	40.77 ± 9.67 40.99 ± 9.26	① < ②	0.022	0.882
Age	①18 ~ 29y ②30 ~ 39y ③40 ~ 49y ④≥ 50y	63 52 36 28	35.2 29.1 20.1 15.6	40.00 ± 9.14 41.63 ± 8.89 41.04 ± 10.83 41.56 ± 8.99	① < ② ③ < ④	0.346	0.792
Marital status	①Married ②Unmarried	127 52	70.9 29.1	41.43 ± 9.41 39.71 ± 9.21	② < ①	1.241	0.267
Educational level	①Junior high school and below ②High school ③Bachelor degree or above	69 61 49	38.5 34.1 27.4	41.88 ± 8.93 39.57 ± 8.74 41.28 ± 10.62	② < ① ③ < ①	1.036	0.357
Residential status	①Living alone ②Living with spouse or family ③Living with friends or others	6 169 4	3.4 94.4 2.2	39.38 ± 8.83 41.04 ± 9.43 38.75 ± 8.60	③ < ② ② < ①	0.200	0.819
Know relevant knowledge of COVID-19	①Yes ②No	168 11	93.9 6.1	41.02 ± 9.53 39.55 ± 9.36	② < ①	0.255	0.614
Main channels for acquiring relevant knowledge	①Official platform ②Unofficial platforms ③Others ④No knowledge of relevant information	137 32 9 1	76.5 17.9 5.0 0.6	40.60 ± 9.41 41.91 ± 9.77 40.69 ± 6.41 56.25	① < ② ③ < ②	1.070	0.363
Contacted with the people have been to Wuhan within half a month	①Yes ②No	10 169	5.6 94.4	44.00 ± 10.01 40.75 ± 9.32	② < ①	1.141	0.287
Going out or gathering within half a month	①Yes ②No	38 141	21.2 78.8	42.34 ± 9.70 40.55 ± 9.26	② < ①	1.090	0.298
Wearing a mask when going out	①Yes ②No	171 8	95.5 4.5	40.88 ± 947 41.88 ± 7.13	① < ②	0.085	0.771
Adequate masks at home	①Yes ②No	99 80	55.3 44.7	40.44 ± 8.90 41.53 ± 9.92	① < ②	0.598	0.440
Self-rated of health status	①Very good ②Good ③Fair ④Poor	135 34 8 2	75.4 19.0 4.5 1.1	39.73 ± 8.65 43.16 ± 10.27 48.90 ± 11.72 51.88 ± 2.65	① < ②^*^ ② < ③^*^ ③ < ④^*^	4.477	0.005 ^*^
Level of concern about the epidemic	①Not at all concerned ②Had some concerns ③Very worried	11 100 68	6.1 55.9 38.0	35.34 ± 7.83 40.29 ± 9.02 42.78 ± 9.71	① < ②^*^ ② < ③^*^	3.622	0.029 ^*^
Self-rated of the impact of the epidemic on life	①Not affected ②A little bit affected ③Very affected	7 88 84	3.9 49.2 46.9	35.54 ± 9.10 39.72 ± 8.22 40.93 ± 9.36	① < ②^*^ ② < ③^*^	3.408	0.035 ^*^

* *P < 0.05*.

### Scores of Anxiety Levels of Rural Residents in Hunan During the COVID-19 Epidemic

As shown in Table 2, the mean anxiety scores of rural residents in Hunan during the COVID-19 epidemic was 40.93 ± 9.36, which was higher than the rural residents' anxiety scores during the non-epidemic period (*t* = 14.820, *P* < 0.001), which was also higher than the anxiety scores of healthy Chinese individuals during the non-epidemic period (*t* = 55.098, *P* < 0.001). However, compared with the anxiety scores of rural residents across China during the epidemic, rural residents in Hunan had lower anxiety scores (*t* = −4.375, *P* < 0.001). In terms of anxiety detection rate, only the difference between rural residents in Hunan during the epidemic and healthy people before the epidemic was statistically significant (χ^2^ = 6.644, *P* = 0.010).

**Table 2:** Comparison of the anxiety level of rural residents in Hunan during the epidemic and different populations in different periods.

		**Total number of sample**	**Mean (SD)**	***t***	***P*-value**	**Number of anxiety detected (%)**	**χ^**2**^**	***P*-value**
Anxiety level of residents in different regions during the epidemic				−4.375	<0.001		3.708	0.054
	Rural residents in Hunan Province	179	40.93 ± 9.30			32(17.9)		
	Rural residents across China	1,029	43.99 ± 8.55			398(38.7)		
Anxiety level of rural residents in different periods				14.820	<0.001		0.730	0.393
	During the epidemic	179	40.93 ± 9.30			32(17.9)		
	During non-epidemic period	778	30.56 ± 5.94			119(15.3)		
Anxiety level of rural residents in Hunan Province during the epidemic and healthy people during non-epidemic period				55.098	<0.001		6.644	0.010
	Rural residents in Hunan province during the epidemic	179	40.93 ± 9.30			32(17.9)		
	Healthy people during non-epidemic period	1,158	29.78 ± 10.07			129(11.1)		

### Factors Influencing Rural Resident' Anxiety in Hunan During the COVID-19 Epidemic

The results showed that self-rated health status, level of concern about the epidemic, and self-rated impact of the epidemic on one's life had statistically significant effects on anxiety scores (*P* < 0.05, as shown in Table 1).

### Hierarchical Regression Analysis of the Influencing Factors of Rural Residents' Anxiety Score in Hunan During the COVID-19 Epidemic

Hierarchical regression analysis was used to analyze whether the gradual addition of self-rated health status, level of concern about the epidemic, and self-rated impact of the epidemic on one's life could improve the model's prediction level of the standard anxiety score.

The final model included three variables: self-rated health status, level of concern about the epidemic, and self-rated impact of the epidemic on one's life, which was statistically significant, *R*^2^ = 0.113, *F*(3, 175) = 7.465 (*P* < 0.001), adjusted *R*^2^ = 0.098, which explained 11.3% of the total variation in the anxiety scores of rural residents in Hunan during the COVID-19 epidemic.

Self-rated health status was added into Model 1 and the *R*^2^ value of Model 1 was 0.070, *F*(1, 171) = 13.304 (*P* < 0.001). After adding level of concern about the epidemic, the *R*^2^ value of Model 2 increased by 0.035, *F*(1, 176) = 6.890 (*P* < 0.01), which was statistically significant. Based on Model 2 and adding self-rated impact of the epidemic on one's life, the *R*^2^ value of Model 3 increased by 0.009, *F*(1, 175) = 1.678 (*P* = 0.197 > 0.05), which was not statistically significant. The specific results are shown in Table 3.

**Table 3:** Hierarchical regression analysis of the influencing factors of rural residents' anxiety score in Hunan during the epidemic of COVID-19.

**Variables**	**Model 1**		**Model 2**		**Model 3**	
	**B**	**Standardized coefficients B**	**B**	**Standardized coefficients B**	**B**	**Standardized coefficients B**
Interpolation	35.612^***^		28.732^***^		26.652^***^	
Self-rated of health status	4.050^***^	0.264	3.997^**^	0.262	3.935^***^	0.257
Level of concern about the epidemic			2.997^**^	0.187	2.032	0.127
Self-rated of the impact of the epidemic on life					1.810	0.110
*R*^2^	0.070		0.105		0.113	
*F*	13.304^***^		10.319^***^		7.465^***^	
Δ*R*^2^	0.070		0.035		0.009	
Δ*F*	13.304^***^		6.890^**^		1.678	

*N = 179*,

** *P < 0.01*,

*** *P < 0.001*.

## Discussion

### Anxiety Status of Rural Residents in Hunan During the COVID-19 Epidemic

The results of this survey show that the anxiety score of rural residents in Hunan during the COVID-19 epidemic was higher than that of healthy Chinese (26) and rural residents across China (27) during the non-epidemic period. Our research found that the detection rate of anxiety among rural residents in Hunan was 17.88%, while one Turkish study reported that the detection rate of anxiety in people was 45.1% (28) while a study on American adults found a rate of 38.41% (29). The reason for this difference may be that our research was carried out in February, while their research was mainly carried out in April 2020. At this time, COVID-19 was spreading around the world and the epidemic was worsening. Due to the strong contagion, long incubation period, wide spread, and rapid progress of COVID-19, the epidemic seriously threatens people's health and safety, and people will inevitably experience negative emotions such as anxiety and fear when facing the epidemic (30). However, it is worth mentioning that because of the specific geographical location of Hunan (near Hubei Province, the province where the epidemic first broke out), before the survey, researchers predicted that rural residents in Hunan Province may have higher levels of anxiety, but our results show that during the COVID-19 epidemic, rural residents' anxiety scores in Hunan were lower than national rural residents' anxiety scores (43.99 ± 8.55), as investigated by Wang (31). This may be related to the fact that the Chinese government immediately locked down Wuhan and the entire Hubei Province after the outbreak of the epidemic to contain the pandemic, so that the epidemic did not break out in large areas in Hunan Province, and the growth rate of the epidemic gradually decreased; thus, most residents in the vicinity of the epidemic became increasingly confident.

### Factors Influencing the Anxiety of Rural Residents in Hunan During the COVID-19 Epidemic

#### Self-Rated Health Status

Residents who considered themselves to be in poor health had higher anxiety scores than residents who considered their health to be good, and their average anxiety score reached the anxiety cutoff. Metacognition theory posits that self-centered consciousness activities are positive feedback and adjustments to consciousness (32). Therefore, residents who believe they are in good health will be confident in resisting attacks of COVID-19, while residents who believe they are in poor health may think they will be more susceptible to the virus and if they are infected, have no confidence in defeating it, so their anxiety scores would be higher than those who think they are in good health. Studies (33, 34) have shown that emotional therapy has a positive effect on easing the public's negative emotions during the epidemic; therefore, self-positive suggestion may relieve residents' anxiety.

#### Level of Concern About the Epidemic

The higher their level of concern about the epidemic, the higher the residents' anxiety score. Studies by Li (35) and Zhang (24) also showed that the degree of concern about the epidemic is a factor influencing anxiety among different groups of people during the COVID-19 epidemic. Several factors are likely to induce anxiety among residents, including the number of confirmed cases and deaths from COVID-19 that continue to rise daily, lack of knowledge of the disease, surrounding people who may have been infected with the virus, and the lack of anti-coronavirus specific drugs (36), among other reasons. Cai (37) believed that during the epidemic, people only know what COVID-19 is but do not know how to control it, which is not sufficient to reduce its effects on their psychological problems. The key is to know how to prevent and control COVID-19. Leung et al. (38) found that publicizing measures for disease prevention and eliminating the spread of rumors can reduce public anxiety. The results of this survey showed that most people acquired relevant knowledge of COVID-19 through an official platform. Therefore, it is necessary to strengthen the dissemination of disease information and prevention measures on official platforms.

#### Self-Rated Impact of the Epidemic on One's Life

The greater their self-rated impact of the epidemic on one's life, the higher the residents' anxiety scores. The population density in rural areas is smaller than that in cities or towns, and houses are mostly single-family houses, so rural residents can have a wide range of activities in a relatively safe and isolated environment. Some rural residents usually work in fields or farms and rarely go to densely populated areas, so they feel that the epidemic has little impact on their lives and have low anxiety scores. However, some rural residents need to go to cities to earn a living or need to be in contact with others at work; during the epidemic, their financial and living pressures have greatly increased due to work restrictions, so their anxiety scores are high. For these individuals, it is necessary to actively solve practical problems such as the delayed resumption of work and limited social activities (39).

## Limitations

Due to the difficulties and limitations in collecting data during the epidemic, random sampling was not conducted in this survey, and the nature of the sample may limit the generalizability of the results. In addition, due to the use of online questionnaires, the survey only included people who can operate WeChat proficiently. Elderly and rural residents who do not use WeChat were not included. Further longitudinal studies should be conducted to better investigate the psychological status of and interventions for rural residents more deeply.

## Conclusion

COVID-19 is having a huge impact on the life and the psychology of rural residents in Hunan Province, China. During the epidemic, their anxiety rate was high. This was a particular phenomenon of residents in a specific period; we need to pay enough attention to it and seek positive solutions to deal with it ingeniously. The results showed that self-rated health status, level of concern about the epidemic, and self-rated impact of the epidemic on one's life are the main factors that affect how anxious rural residents are. It is necessary to increase publicity about COVID-19 prevention measures and provide positive psychological guidance to alleviate rural residents' anxiety. Timely announcements about the epidemic, vigorous publicity about epidemic prevention measures, and the support of all sectors of society will give people around the world greater confidence and strength to overcome the epidemic (40).

## Data Availability Statement

The raw data supporting the conclusions of this article will be made available by the authors, without undue reservation.

## Ethics Statement

The studies involving human participants were reviewed and approved by The Ethics Committee of the Xiangya Nursing School, Central South University. Written informed consent for participation was not required for this study in accordance with the national legislation and the institutional requirements.

## Author Contributions

YZ and Y-pC were the primary investigator of the study, did statistical analysis and wrote this paper. JW and YD helped conduct the study and revised the manuscript. DP and LZ helped supervised the survey and checked the data. All authors contributed to and approved the final manuscript.

## Conflict of Interest

The authors declare that the research was conducted in the absence of any commercial or financial relationships that could be construed as a potential conflict of interest.
